# Predicting and understanding non-adherence in chronic disease: cross-cohort validation and structural equation modeling of the SPUR 6/24 tool

**DOI:** 10.1038/s41598-025-17866-6

**Published:** 2025-09-26

**Authors:** Kevin Dolgin, Reem Kayyali, Joshua Wells, Chao Wang

**Affiliations:** 1 IAE Paris-Sorbonne Business School 8 Bis, Rue de La Croix Jarry, 75013 Paris, France; 2 Department of Pharmacy, Kingston University, Kingston Hill, Kingston Upon Thames, Surrey KT2 7LB UK; 3 Honorary Lecturer, Kingston University, Kingston Hill, Kingston Upon Thames, Surrey KT2 7LB UK; 4 Health & Social Care Statistics, Faculty of Health, Science, Social Care and Education, Kingston University, Kingston Hill, Kingston Upon Thames, Surrey KT2 7LB UK

**Keywords:** Medication adherence, Structural equation modeling, Behavioral drivers, Patient-reported outcomes measures (PROM), Patient-reported adherence measures (PRAM), Health behavior, SPUR tool, Cross-cohort analysis, Health services, Public health

## Abstract

The SPUR tool measures the risk of non-adherence for patients with chronic disease, as well as measuring the relative importance of thirteen behavioral drivers contributing to that risk. Over a period of four years, five different cohorts of patients in three countries and three different pathologies were studied to contribute to the elaboration and refinement of two patient-reported adherence measures: SPUR 6 and SPUR 24. This article examines the results of retrofitting of both of these tools to earlier patient cohorts as well as analyzing the pooled dataset via the use of both tools in order to further study the predictive potential of both. A further analysis was carried out using structural equation modeling both to test the structural validity of the SPUR tools and to examine both indirect and direct influence of the thirteen drivers on patient behavior.Direct comparisons of the SPUR tools to other patient-reported adherence measures across datasets and across the pooled dataset was carried out by analysis of Spearman’s ranked correlation coefficients. The structural equation modeling was carried out using path analysis based on the decision-making schema hypothesized in the foundational SPUR article.The retrofitted analysis and the pooled data analysis both support the use of SPUR 6 and SPUR 24 to assess the risk of non-adherence of patients with chronic disease with respect to other widely used patient reported adherence measures. The structural equation modeling reinforced the hypothesis that the social and psychological drivers of SPUR have a significant indirect impact on non-adherence risk via the rational and usage drivers as well as their direct impact on non-adherence risk.SPUR 6 and SPUR 24 have demonstrated predictive value in assessing the risk of patient non-adherence as compared to their predecessors as well as to other widely-used patient adherence measures, across countries and pathologies. The social and psychological drivers of SPUR seem to drive behavior largely through their influence on rational and usage factors, indicating a cognitive rationalization process . These insights have direct implications for communication strategy towards patients in efforts to enhance medication adherence.

## Introduction

The SPUR PRAM (patient-reported adherence measure) was developed over 2021 and 2022 via a series of studies in Europe and the United States. Based on the initial SPUR theoretical framework first published in Patient Preference and Adherence^1^, it was designed to allow for the assessment of non-adherence risk for patients with chronic disease via an interactive, digital tool that further identifies the specific drivers of that risk.

SPUR is based on 13 specific drivers, grouped into four categories (Table 1): Social, which describes the degree to which the illness and the treatment affects the patient’s immediate social interactions and their perception of their role in society; Psychological, which groups drivers concerning the patient’s degree of psychological reactance, their ability to reconcile their disease and treatment with their self-concept and the degree to which they discount future benefits; Usage, consisting of drivers relating to forgetfulness, ability to access treatment, their ability to follow the treatment, and the financial burden it represents; and rational, representing the patient’s assessment of the relative risks and benefits of the treatment given their appreciation of the disease gravity and severity, in line with the Health Belief Model^2^.

**Table 1:** SPUR driver descriptions and abbreviations.

Category	Driver	Abbreviation	Description
Social	Social Immediate	Si	The degree to which the illness and the treatment disrupt the patient’s immediate social environment, centering on family and friends
	Social Societal	Ss	The degree to which the patient feels that the disease or the treatment disrupts their place in society
Psychological	Reactance	Pr	The patient’s tendency to reject authority
	Discounting of future benefits	Pt	The degree to which the patient focuses on short-term as opposed to long-term effects
	Identity	Pi	The degree to which the patient has difficulty incorporating the reality of their disease and their treatment into their concept of self
Usage	Forgetfulness	Ufo	The extent to which the patient has difficulty remembering to follow their treatment
	Access	Ua	The degree to which the patient has difficulty obtaining what is needed to follow the treatment
	Capability	Uc	The degree to which the patient finds it difficult to administer or follow their treatment plan
	Financial	Ufin	The degree to which the patient feels that following the treatment represents a financial burden to them
Rational	Disease gravity	Rdg	The extent to which the patient’s perception of the gravity of the disease represents a barrier to their adherence to treatment
	Disease susceptibility	Rds	The degree to which the patient’s impression of their susceptibility to negative consequences affects their adherence
	Treatment Benefit	Rtb	The degree to which the patient’s perception of the treatment’s benefits affects their adherence
	Treatment Problems	Rtp	The degree to which the patient’s perception of problems with the treatment affects their adherence

The tool itself is designed to provide an assessment of patients’ risk of non-adherence while at the same time providing insights into the causes of that risk, using measures of the thirteen drivers. After four years of study and development, sufficient data has been collected to permit analysis of pooled data of several cohorts, across three countries and four pathologies. This analysis allows the current, revised version of the tool to be applied to earlier cohorts which were assessed using previous versions as well as to the pooled data to further test the validity of the tool’s ability to identify non-adherence risk. The larger pooled data set also permits the use of structural equation modeling (SEM) to provide deeper insights into the interplay between the different behavioral drivers. This paper discusses the results of these analyses across five cohorts of patients.

## Development of the tool

After publication of the initial theoretical framework of SPUR^1^, a review of existing patient-reported adherence measures (PRAMs) was carried out and items were derived to measure the thirteen drivers. These items were tested via interviews with French, British, US and Chinese patients and 45 items were retained^3^. Over the course of further development of the tool, two different approaches were examined, both using the same 45 items. In the UK, factor analysis was used to refine the tool to a 27-item version (SPUR 27), initially using a cohort of 378 patients with type 2 diabetes^4^. SPUR 27 was then tested with a 100-patient cohort with COPD^5,6^ and was further tested for its ability to predict hospital re-admission via a follow-up analysis on the initial diabetes cohort^5,6^.

In parallel to the work in the UK, the initial 45-item SPUR questionnaire was analysed with a cohort of 501 people with diabetes in the United States using a partial credit model (PCM). Partial least-squares discriminant analyses (PLS-DA) was then used to map the items to the theoretical framework^7,8^. This was further refined using a cohort of 245 French patients^7,8^ in which the PCM model became the basis for a Raasch model via principle component analysis. This allowed the development of a more flexible model of SPUR, in which a 6-item initial questionnaire proved highly predictive of non-adherence while a more complete 24-item version (including the original six items) provided heightened predictive value while further allowing more precise measurement of the underlying behavioral drivers. This SPUR 6/24 version contained almost all of the items used in SPUR27. SPUR 6/24 was retained as the suggested version of SPUR to be used in clinical settings.

These studies collectively demonstrate the potential of SPUR to help assess patient behavioral risk and to identify the drivers of that risk. A further cohort of 571 patients in the United States living with cardiovascular disease was recruited in 2022 to assess the use of SPUR in this pathology and to examine its potential to identify patients living with mental illness^9^(Results were presented in a poster at ESPACOMP 2022, a paper is currently pending.) The data from this cohort are currently being studied and will be the subject of a paper to be submitted for peer review shortly. This cohort was included in the pooled analysis presented in this paper.

All of the patients in all of the cohorts analyzed responded to all 45 of the initial SPUR items. As such, whether they were analyzed using SPUR 45, SPUR 27 or SPUR 6/24 it was possible to retrospectively calculate their SPUR 6/24 scores. As SPUR 6/24 has been retained as the standard SPUR version, it is therefore of interest both to re-assess all the cohorts using this same tool, as well as analyzing the pooled data from all five cohorts to consider the performance of SPUR 6/24 across a population that is heterogeneous both in terms of culture and pathology.

The patients studied in the five cohorts (Table 2) described above represent three different therapeutic areas across three countries. Each of these cohorts of patients responded to SPUR items as well as being presented with a number of other PRAMs. All papers used validated measures according to approvals sought. The current analysis compares SPUR 6/24 scores to the SPUR scores originally calculated as well as analyzing SPUR 6/24 across the pooled data of all five cohorts with respect to the other measures applied.

**Table 2:** composition of cohorts and validated measures used.

	^7,8^ USA, DT2	^7,8^ France, DT2	^4^ UK, DT2	^5,6^ UK, COPD	Dolgin, Lee 2025 (pending) USA, Cardiovascular
Pathology	Type 2 diabetes	Type 2 diabetes	Type 2 diabetes	COPD	Hypertension
Number of patients	501	245	378	200	571
NAR6 (SPUR)	x	x	x	x	x
NAR24 (SPUR)	x	x	x	x	x
HbA1c		x	x		
MPR			x	x	
MMAS-8	x				x
MARS		x	x		
BMQ-specific	x	x	x		x

## Methodology

The primary objective of this comparative analysis is to compare SPUR 6/24 scores to the originally calculated SPUR scores and to analyze SPUR 6/24 scores across the pooled data of all five cohorts in relation to other applied measures. This approach aims to enhance precision in risk determination, refine the understanding of individual behavioral drivers, and reduce the overall testing burden.

The study utilizes a retrospective, cross-sectional design, leveraging data collected from five independent cohorts. SPUR 6/24, a streamlined version of the SPUR model first elaborated in^7,8^ is evaluated for its performance in predicting health outcomes and identifying behavioral drivers. The analysis involves comparing SPUR 6/24 scores to the other variants of SPUR scores originally used and examining their relationships with other validated measures administered within each cohort. As the items used in all of the original studies included the 24 items necessary to calculate SPUR 6/24 (as well as other SPUR items not retained) the calculation of SPUR 6/24 was carried out retrospectively simply by applying the algorithms described in^7,8^.

### Data sources

Data for this analysis were pooled from five separate cohorts representing diverse populations and health conditions. Each cohort included a set of variables capturing demographic, clinical, and behavioral data alongside SPUR scores and other relevant measures. The SPUR 6/24 model is derived from the same questionnaire but includes a reduced number of items to alleviate participant burden while preserving predictive utility.

### Variables

- SPUR Scores: Both SPUR6 and SPUR24 scores were calculated for each participant.
- The use of SPUR 24 also allowed values to be calculated for the 13 behavioral drivers described in Table 1 for all cohorts.
- Other Measures: Cohorts included additional validated instruments that assessed health outcomes, behavioral traits, and risk factors.
- Demographics and Clinical Data: Age, gender, comorbidities, and other relevant variables were incorporated to contextualize findings.

### Statistical analysis

1. Comparison of SPUR 6/24 and earlier SPUR Scores:

Spearman’s rank correlation coefficients were used to assess the relationship between SPUR 6/24 and SPUR scores generated using previous SPUR scoring variants. This analysis aimed to determine the strength and direction of monotonic associations between the different scoring systems.

2. Analysis across pooled cohort data:

Descriptive statistics summarized the distribution of SPUR 6/24 scores across cohorts. Spearman’s rank correlations were then also applied to examine the associations between SPUR 6/24 scores and other applied measures across pooled cohort data. These were compared with the correlations of other validated PRAMs for the same measures.

### Ethical considerations

This study involved secondary analysis of anonymized observational data drawn from five previously approved and ethically reviewed patient cohorts. No new data were collected for the current analysis, and further ethical review was therefore not deemed necessary. Below are the details of the original ethical approvals for each cohort:

- **UK Diabetes Cohort:** Ethical approval was obtained for the community arm from the Kingston University Research Ethics Committee (Ref: 1819.081.1) and for the hospital arm (VMATT2) from the NHS Integrated Research Application System (IRAS ID: 270,768) via Research Ethics Committee review (Ref: 19/NW/0685). Informed consent was obtained from all participants.
- (Wells et al., *BMJ Open*, 2022: 10.1136/bmjopen.058467)
- **UK COPD Cohort:** This follow-up study (VMATC) received ethics approval from the NHS Health Research Authority via IRAS (ID: 285,590) and Research Ethics Committee (Ref: 20/NW/0485) in January 2021. Informed consent was obtained from all participants. (Wells et al., *Patient Preference and Adherence*, 2023: 10.2147/PPA.S394538)
- **US Diabetes Cohort:** *Mechanical Turk.* Approved by the Institutional Review Board (IRB) at the University of Michigan. Informed consent was obtained from all participants. (de Bock et al., *Current Medical Research and Opinion*, 2022: 10.1080/03007995.2021.2010437)
- **French Cohort:** Approved by the French Comité de Protection des Personnes (CPP). Informed consent was obtained from all participants. (de Bock et al., *Patient Preference and Adherence*, 2022: 10.2147/PPA.S354705)
- **US Hypertension Cohort:** Data was gathered via *Mechanical Turk*. The University of Mississippi IRB reviewed the protocol (SPUR Antihypertensive Adherence Validation Study, Protocol #22x-101) and determined it to be exempt under 45 CFR 46.101(b)(2). Participants gave informed consent.

All datasets were anonymized prior to analysis and handled in accordance with relevant data protection regulations.

### Software

All statistical analyses were performed using R (version 4.3.0).

## Results of retrospective analysis

When the most recent SPUR 6/24 scoring was applied to the initial cohorts individually, SPUR 6/24 proved to be correlated significantly both to the PRAMs and to the clinical outcome measures. Table 3 shows the minimum and maximum Spearman’s rank correlations for SPUR6 and SPUR24 with respect to each of the variables studied across the five cohorts. Note that *p* < 0.01 in all cases, except where given.

**Table 3:** Spearman’s ranked correlation, absolute values across cohorts. P < 0.01 except where indicated.

	HbA1c	MPR	MMAS-8	MARS	BMQ-specific
# of cohorts present	2	2	2	2	4
SPUR6 minimum *r*	0.12 (*p* = 0.05)	0.12 (*p* = 0.02)	0.33	0.26	0.33
SPUR6 maximum *r*	0.16	0.33	0.42	0.26	0.56
SPUR 24 minimum *r*	0.11 (*p* = 0.08)	0.16	0.42	0.22	0.50
SPUR 24 maximum *r*	0.18	0.32	0.59	0.41	0.65

It is worth noting that in all cases, both SPUR measures had a higher correlation to the two non-PRAM measures (MPR and HbA1c) than the other PRAMs, and in all cases but one, SPUR24 had a higher correlation to each of the individual PRAMs than any of the other PRAMs (Table 4). The correlations to other variables were not statistically different to that of the earlier versions of SPUR, although SPUR27 was more closely correlated to MPR in the UK diabetes study.

**Table 4:** Spearman’s rank correlation, absolute values. p < 0.01 except where indicated. Correlations of 0 indicate no statistical significance (p > 0.10).

	MPR	BMQSPEC	MMAS8	MARS	SPUR6	SPUR24
HBA1C	0	0.13 (p = 0.03)	NA	0	0.15	0.16
MPR	-	NA	NA	0	0.16	0.18
BMQSPEC	-	-	0.29	0.40	0.34	0.53
MMAS8	-	-	-	NA	0.32	0.33
MARS	-	-	-	-	0.27	0.29
SPUR6	-	-	-	-	-	0.80

For the pooled data set, the correlations are given in Table 4, including the correlations between all variables.

In all cases, SPUR 24 had a higher correlation to each of the studied variables than the other PRAMs measured. In all cases but one, SPUR6 likewise outperformed the other PRAMs and in all cases, both SPUR results had significant correlations to the other studied variables.

The variability of the correlations across the studies for all variables is most evident in the case of MARS for the PRAMs and notably, for MPR in the two studies in which this was measured. In the first UK study (Type 2 Diabetes), the data necessary to calculate 6-month MPR was gathered by collecting data over the telephone from pharmacists, whereas in the second study, it was gathered directly by the lead author, who examined electronic health records. Furthermore, the data collection in the first case was interrupted by COVID lockdowns, leading in reality to two separate cohorts: pre-lockdown and post-lockdown. During lockdown, most medications were provided using regular postal deliveries irrespective of patients’ medicines use. In the case of this study, MPR was not correlated to HbA1c. In neither case were the constituents of adherence according to the ABC taxonomy calculated^10^. Correlations to MPR for all PRAMs were relatively weak for this study, with SPUR24 having the highest correlation. In the second UK study, SPUR correlations to MPR were significantly higher (other PRAMs were not administered for this study). This underscores the importance of rigorous methodology when using MPR.

## Structural equation modeling results

The algorithm of the SPUR tool considers the contribution of the items both to the subject’s risk of non-adherence and to the scores of the behavioral drivers^7,8^. Analysis of the pooled data set demonstrates that there are further dynamics at play with respect to the influence of the various drivers on each other. Notably, while there is a general tendency for patients to have positive scores in multiple drivers as opposed to expressing single drivers, a pattern emerges in which social (S) and psychological (P) drivers tend to be more strongly associated with Usage (U) and Rational (R) drivers than with each other, while the same is true for Usage (U) and Rational (R) drivers. As illustrated in Table 5, the odds of S or P drivers affecting R or U drivers is considerably greater than the odds of S and P drivers being expressed together, or R and U drivers being expressed together. The McNemar’s p value for these “indirect” vs. “direct drivers” is < 0.01.

**Table 5:** Odds ratios of driver category interactions.

Variable	Reference	OR	95% CI	*P*-value
S or P	U or R	6.53	5.29–8.06	< 0.01
S drivers	P drivers	2.32	1.88–2.86	< 0.01
U drivers	R drivers	3.96	3.24–4.82	< 0.01

**Fig. 1:**
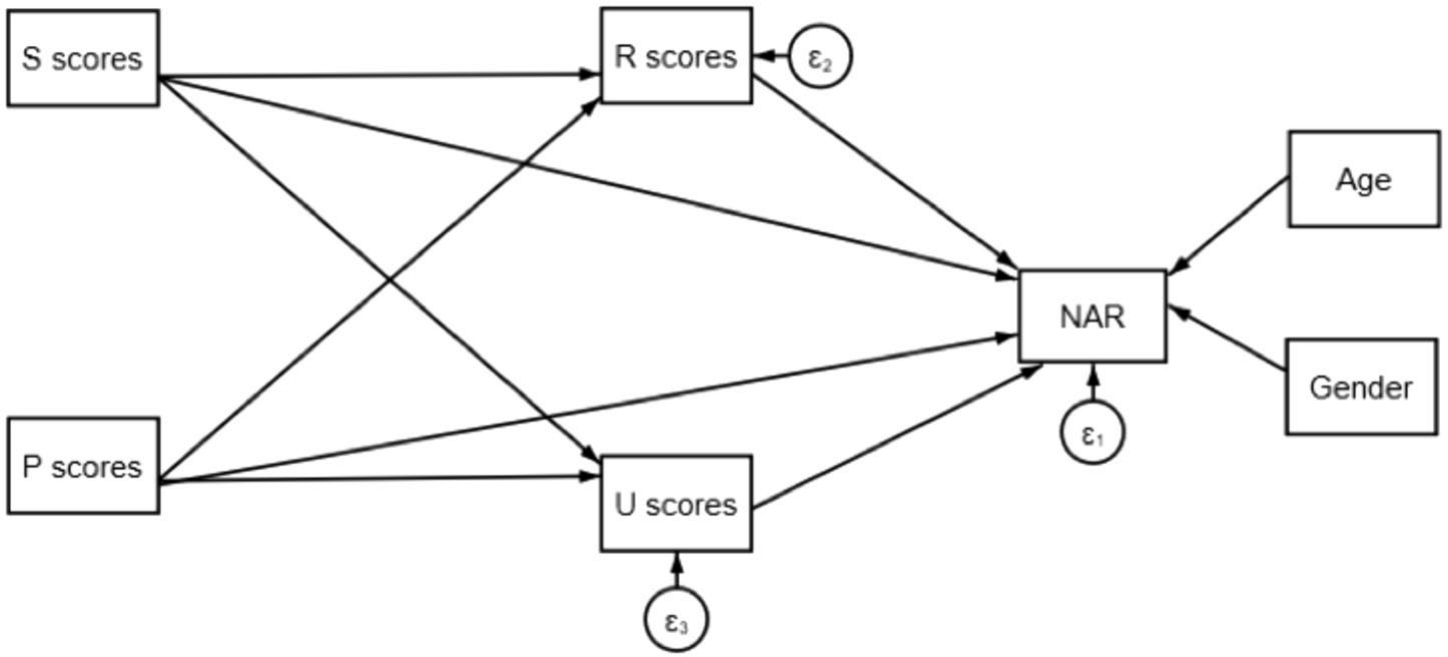
Hypothesized path diagram used in the structural equation model.

In order to examine this phenomenon more completely, a structural equation modelling (SEM) analysis was undertaken using CB-SEM (covariance-based SEM). SEM on the pooled data allows us to examine both the direct impact of each driver on non-adherence risk (NAR) as well as the driver’s indirect impact via its influence on other drivers. The measures for each driver were calculated using the methodology described in de Bock et al.^7,8^. The SEM tested the hypothesis that psychological and social drivers have important indirect impact on NAR via their influence on rational and usage drivers, as well as direct impact (Fig. 1).

Because the data was clustered (as it originated in five distinct cohorts), cluster-robust standard errors were used to account for the correlations within cohorts / clusters. As such, the usual likelihood-based goodness of fit statistics were not valid (such as pseudo-R^2^ and AIC). We used root mean squared residual (SRMR) and overall r^2^ as a measure of model quality. The SEM resulted in SRMR = 0.068, while R-squared = 0.835, indicating a good model fit^9^.

Table 6 shows the direct, indirect, and total impact of each driver on NAR according to the SEM results and indicates the *p*-value of the influence (For all results p < 0.01 except where indicated, no value indicates no significant impact). Drivers are ranked according to their total impact on NAR.

**Table 6:** Direct, indirect and total impact of drivers on NAR.

Influence on NAR	Direct	Indirect	Total
Rds	**-0.02 (p < 0.05)**	**0.00**	**-0.02 (p < 0.05)**
Pt	**0.04**		**0.04**
Uc	**0.05**	**0.00**	**0.05**
Ua	**0.05**	**0.00**	**0.05**
Rtp	**0.06**	**0.00**	**0.06**
Ufin	**0.08**	**0.00**	**0.08**
Ss	**0.00**	**0.08**	**0.08 (p < 0.05)**
Ufo	**0.10**	**0.00**	**0.10**
Rtb	**0.12**	**0.00**	**0.12**
Si	**0.09 (p < 0.05)**	**0.03**	**0.12**
Pi	**0.08**	**0.07**	**0.14**
Rdg	**0.16**	**0.00**	**0.16**
Pr	**0.12**	**0.20**	**0.31**

As posited, social and psychological drivers display both direct and indirect impacts on NAR, whereas rational and usage drivers influence NAR directly. The one exception to this is the Pt driver, which does not have any indirect impact that is statistically significant to any drivers at *p* < 0.01, although the influence of Pt on Rtb, while minor, is significant at *p* < 0.05.

Further analysis showed that the social societal (Ss) driver has very little direct impact on NAR. Instead, it affects NAR primarily via its influence on all of the rational and usage drivers. As can be seen in Table 5, the Ss driver has statistically significant influence (p < 0.01) on all the rational and usage drivers, with a particular influence on Ufin, or the indicator of financial burden. The other social driver, social immediate (Si), has far lower indirect influence, affecting only Rdg and Ufin at p < 0.01 and with a slight influence on Rds at p < 0.05.

Two of the three psychological drivers have strong indirect influence. Psychological, identity (Pi) has significant direct impact on NAR, as well as roughly the same level of indirect impact via its influence of three rational drivers and two usage drivers, while psychological, reactance (Pr) has both significant direct impact on NAR, while even greater indirect impact, affecting all rational and usage drivers. When it is activated for a patient, it represents the most significant of the 13 drivers, as seen in Table 7

**Table 7:** Influence levels of P and S drivers on R and U drivers. For all results p < 0.01 except where indicated.

	Rdg	Rds	Rtb	Rtp	Ua	Uc	Ufin	Ufo
Pr	0.287	0.318	0.319	0.423	0.268	0.432	0.255	0.381
Pi	0.117	0.137	0.127		0.237	0.181		0.11(p < 0.05)
Pt			0.035 (p < 0.05)					
Ss	0.096	0.031	0.113	0.131	0.146	0.101	0.211	0.134
Si	0.059	0.02(p < 0.05)					0.074	

Note that age and gender were also tested and were found to not exhibit significant direct or indirect impact on NAR.

## Discussion

With respect to the use of SPUR 6/24 across previous cohorts and across the pooled data, this analysis demonstrates the ability of the tool to provide a useful indication of risk of non-adherence. The robustness of the results across pooled data representing patients in three different pathologies and three different countries indicates a promising degree of agnosticism across cultures, environments, and therapeutic areas.

While some of the correlations observed between SPUR scores and the other PRAMs or clinical outcomes fall in the moderate range (e.g., 0.3–0.4), such values are consistent with expectations in behavioral research, where multiple factors influence adherence. Importantly, SPUR – particularly SPUR24 – generally showed stronger correlations with both PRAMs and objective adherence outcomes (e.g., MPR, HbA1c) than the other PRAMS. This suggests that SPUR captures distinct and meaningful dimensions of patient behavior that contribute to its predictive utility. It is of interest that both SPUR6 and SPUR24 are most closely correlated with the BMQ Specific driver than with either MARS or MMAS-8 (see Table 4). Both MMAS-8 and MARS are “first-generation” PRAMs in that they directly query the patient’s behavior, with items such as “Did you take your medication yesterday?” or “I forget to take my medications”. Both SPUR and BMQ attempt to derive behavior via more indirect questions, both to avoid response bias and to gain an understanding of behavioral drivers. As such, the higher correlation between them is in line with their underlying design philosophy.

This may indicate that the SPUR scores, as well as the BMQ scores, are less representative of actual behavior than the more direct first-generation PRAMs. However, in those cohorts in which actual adherence was measured via MPR or clinical endpoints (HbA1c), SPUR scores – and BMQ scores – were more closely correlated to these objective measures than was MARS5 (note that the studies that included MMAS-8 did not include either of these objective measures). It is of interest to determine whether attitudinal PRAMs such as SPUR and BMQ are more divorced from actual behavior due to their less direct questioning or whether they are in fact more closely correlated to actual behavior thanks to the decreased response bias that such a questionnaire strategy provides. Our results indicate that the latter hypothesis has merit, but more research is needed.

With respect to the SEM analysis of the behavioral drivers, the initial hypothesis regarding the presence of both direct and indirect impact of the P and S drivers appears valid. This result supports previous research. For example, the importance of reactance as an influence on non-adherence is supported in the literature. In 2000, Fogarty and Youngs outlined the theoretical constructs behind such a supposition, including the relationship between reactance and locus of control, although their limited experimental design failed to support their hypotheses^11^. In 2011, Gérard Reach described a strong link between obedience and medication adherence in patients with type 2 diabetes^12^ and in 2014, De las Cuevas, Peñate and Sanz documented a significant link between measures of psychological reactance and adherence^13^.

Our analysis provides further evidence for that link while indicating that the impact of reactance may not be self-evident. Two other SPUR drivers, perception of disease gravity (Rdg) and the perception of treatment benefit (Rtb), have higher direct influence scores on NAR than does reactance, but when total impact is taken into account, reactance overshadows all the other drivers significantly. It does so by the way it drives the other, more “rational” and “practical” drivers.

A similar phenomenon is apparent in the Ss and Pi drivers. In the former case, the social societal (Ss) driver *only* influences NAR via its impact on R and U drivers and in the case of Pi, its total influence is considerably greater than its direct influence, making it second only to reactance and disease gravity in terms of its total impact on NAR.

The Ss and Pi drivers are closely related to identity. Pi is designed to give insight into the patient’s narrative, in line with narrative identity theory, whereas Ss is linked to elements of social identity theory. If this indirect impact is linked to concepts of self-identity, it is not surprising to see that the social, immediate score is less prone to indirect impact, as it pertains more to the day-to-day experience of the patient and less to their perception of themselves with respect to society as a whole.

This idea of indirect influence coincides well with the experience of many healthcare providers. It is rare that a patient explains their nonadherence by citing their reluctance to do as instructed, or the disconnect they feel between their sense of identity and the concept of being a patient. Patients tend instead to rationalize their behavior, both to healthcare professionals and to themselves, by more practical arguments, such as those represented by the R and U drivers of SPUR, explaining that they do not feel the drug is effective, or that it is difficult to access, or that they dislike its side effects.

It is evident from this analysis that subjects often have several layers of drivers affecting their health behavior, including both direct factors, such as their beliefs about medication and treatment, and deeper, less evident layers that concern things like reactance and identity. It is further evident that these deeper factors not only directly affect behavior, but also strongly influence the stated, direct factors. This includes, for example, forgetfulness, which is influenced both by reactance and social societal drivers (0.381 and 0.134, respectively, p < 0.01 in both cases).

This multi-layered schema for adherence behavior in chronic disease supports both the underlying SPUR behavioral framework, but also, and more explicitly, the common sense model proposed by Hagar et al., and extended since^14^. This model posits the creation of a personal schema regarding the disease and the treatment, influenced by both endogenous and external sources and strongly colored by emotion, with different factors influencing each other to drive behavior.

A key limitation of this study lies in the composition of the cohorts included in the pooled analysis. Three out of the five cohorts, representing 59% of the total population studied, were composed of patients living with type 2 diabetes, which may lead to an overrepresentation of behavioral, psychological, and social dynamics specific to this pathology. Diabetes, particularly type 2, is well-documented to involve a complex interplay of psychological constructs such as identity conflict, health-related distress, and social stigma, which may accentuate the role of psychological and social factors in adherence behavior. While the SPUR model aims to be agnostic across disease types and cultural contexts, the predominance of diabetes cohorts in this dataset may amplify the observed influence of certain drivers—particularly psychological reactance and social societal disruption—relative to what might be observed in other chronic conditions. Although the inclusion of cohorts with COPD and hypertension provides some cross-pathology validation, future research should include broader representation from additional chronic diseases to confirm the generalizability of SPUR’s structure and predictive validity.

## Conclusions

The validity of SPUR 6/24 both to determine the risk of non-adherence and the relative importance of the drivers behind that risk for each patient is reinforced, both in comparison to preceding versions of the tool and via its ability to provide robust results across heterogeneous patient cohorts.

The results of the SEM analysis provide insights not only into the relative impact of different behavioral drivers, but also into their “nested” nature. This has ramifications for the design of behavioral interventions. Tools such as the Theoretical Domains Framework^15^ and the behavior change technique taxonomy^16^ have proved their value in outlining and categorizing the various drivers of patient behavior and the behavior change techniques (BCTs) that are best suited to addressing them. When faced with personalisation of interventions, it is cogent to understand the structure of these drivers for each patient, lest the intervention try to do everything all at once for everyone, therefore reducing the impact of any specific intervention. As such, a behavioral diagnostic tool such as SPUR can help guide the personalisation of the intervention.

If the drivers for any individual patient are taken at face value, our analysis indicates that too much emphasis may be placed on rational and usage factors as expressed by the patient, whereas for some, these factors may themselves be driven by deeper, perhaps less explicitly stated drivers. The patient who expresses reservations – to their healthcare professional or even to themselves – about problems associated with the treatment may also be expressing their underlying reactance, perhaps exacerbated by a poor relationship with their healthcare professional. The patient who seems unconvinced by the real benefits afforded by the treatment may also be reactant, and / or having difficulties stemming from their sense of self, both at the individual and the social level. If these patients are simply provided educational tools touting the very real benefits of the treatment and minimizing side effects, then the underlying influences on these attitudes are not being addressed and the effectiveness of these interventions may suffer. Patients such as these may benefit from a more holistic approach that addresses not only their rational and usage concerns but also the discomfort they may feel with the treatment stemming from their psychological and social factors.

This insight applies not only to the design of specific behavioral interventions but also, potentially, provides some guidance to healthcare professionals who are dealing with chronic patients. It can behoove them not to take at face value the reservations expressed by their patients with respect to rational or practical considerations then simply reassure or educate them on these factors. By all means, if the patient seems to need such information and support then it should be provided, but the healthcare professional would do well in these cases to dig a little deeper and try to understand if the patient’s recalcitrance is *only* the result of rational or practical considerations or if there is perhaps something deeper behind it. In the latter case, the practical and educational support would benefit greatly by a specific understanding of these drivers, in which case greater shared decision making might help the reactant patient and stories of patient testimonials might help the patient with social identity issues… to name only two examples.

On the whole, this analysis underlines the need for a more holistic understanding of the patient’s behavioral profile and a careful choice of behavioral change techniques to help address the needs not only of the “patient” but of the person the patient represents, in all of their complex glory.

## Electronic supplementary material

Below is the link to the electronic supplementary material.

Supplementary Material 1

## Data Availability

The datasets used and/or analysed during the current study are available from the corresponding author on reasonable request.

## Abbreviations

BMQ: Beliefs about medicines questionnaire
BMQSpec: Beliefs about medicines questionnaire, specific
COPD: Chronic obstructive pulmonary disease
HbA1c: Hemoglobin A1C
MARS: Medication adherence report scale
MMAS: Morisky medication adherence scale
MPR: Medication possession ratio
NAR: Non-adherence risk
PRAM: Patient-reported adherence measure
SEM: Structural Equation Modeling
