# Supplementary material for: Predicting and understanding non-adherence in chronic disease: cross-cohort validation and structural equation modeling of the SPUR 6/24 tool
Source: Sci Rep. 2025 Sep 26;15:33216. doi: 10.1038/s41598-025-17866-6 (PMC12475032; doi:10.1038/s41598-025-17866-6)
Supplement: Supplementary file 1 — Supplementary Material 1 [file 41598_2025_17866_MOESM1_ESM.docx]

**Appendix: complete Structural Equation Modeling impact results**

|  | **Direct** | **Indirect** | **Total** |
| --- | --- | --- | --- |
| **NAR** | | | |
| Uc | 0.045^***^ | 0 | 0.045^***^ |
|  | -10.43 | (.) | -10.43 |
|  |  |  |  |
| Ufin | 0.078^***^ | 0 | 0.078^***^ |
|  | -10.46 | (.) | -10.46 |
|  |  |  |  |
| Ua | 0.054^***^ | 0 | 0.054^***^ |
|  | -4.59 | (.) | -4.59 |
|  |  |  |  |
| Ufo | 0.103^***^ | 0 | 0.103^***^ |
|  | -4.94 | (.) | -4.94 |
|  |  |  |  |
| Rtb | 0.118^***^ | 0 | 0.118^***^ |
|  | -14.36 | (.) | -14.36 |
|  |  |  |  |
| Rdg | 0.161^***^ | 0 | 0.161^***^ |
|  | -9.88 | (.) | -9.88 |
|  |  |  |  |
| Rtp | 0.062^**^ | 0 | 0.062^**^ |
|  | -2.7 | (.) | -2.7 |
|  |  |  |  |
| Rds | -0.02 | 0 | -0.02 |
|  | (-1.17) | (.) | (-1.17) |
|  |  |  |  |
| Age | -0.019 | 0 | -0.019 |
|  | (-0.82) | (.) | (-0.82) |
|  |  |  |  |
| gender2 | -0.097 | 0 | -0.097 |
|  | (-0.38) | (.) | (-0.38) |
|  |  |  |  |
| gender3 | -2.605 | 0 | -2.605 |
|  | (-0.97) | (.) | (-0.97) |
|  |  |  |  |
| Si | 0.094^*^ | 0.026^**^ | 0.120^**^ |
|  | -2 | -2.71 | -2.72 |
|  |  |  |  |
| Ss | 0.001 | 0.079^***^ | 0.08 |
|  | -0.04 | -5.97 | -1.83 |
|  |  |  |  |
| Pi | 0.078^***^ | 0.066^***^ | 0.144^***^ |
|  | -11.52 | -3.96 | -6.28 |
|  |  |  |  |
| Pr | 0.116^***^ | 0.197^***^ | 0.313^***^ |
|  | -10.14 | -8.4 | -9.02 |
|  |  |  |  |
| Pt | 0.040^***^ | 0.004 | 0.044^***^ |
|  | -11.77 | -0.41 | -3.83 |
| **Uc** | | | |
| Si | 0.017 | 0 | 0.017 |
|  | -0.5 | (.) | -0.5 |
|  |  |  |  |
| Ss | 0.101^***^ | 0 | 0.101^***^ |
|  | -3.9 | (.) | -3.9 |
|  |  |  |  |
| Pi | 0.181^***^ | 0 | 0.181^***^ |
|  | -5.42 | (.) | -5.42 |
|  |  |  |  |
| Pr | 0.432^***^ | 0 | 0.432^***^ |
|  | -6.76 | (.) | -6.76 |
|  |  |  |  |
| Pt | -0.004 | 0 | -0.004 |
|  | (-0.32) | (.) | (-0.32) |
| **Ufin** | | | |
| Si | 0.074^***^ | 0 | 0.074^***^ |
|  | -4.74 | (.) | -4.74 |
|  |  |  |  |
| Ss | 0.211^***^ | 0 | 0.211^***^ |
|  | -6.06 | (.) | -6.06 |
|  |  |  |  |
| Pi | 0.051 | 0 | 0.051 |
|  | -0.89 | (.) | -0.89 |
|  |  |  |  |
| Pr | 0.255^***^ | 0 | 0.255^***^ |
|  | -10.76 | (.) | -10.76 |
|  |  |  |  |
| Pt | 0.002 | 0 | 0.002 |
|  | -0.08 | (.) | -0.08 |
| **Ua** | | | |
| Si | -0.002 | 0 | -0.002 |
|  | (-0.07) | (.) | (-0.07) |
|  |  |  |  |
| Ss | 0.146^***^ | 0 | 0.146^***^ |
|  | -5.78 | (.) | -5.78 |
|  |  |  |  |
| Pi | 0.237^***^ | 0 | 0.237^***^ |
|  | -4.05 | (.) | -4.05 |
|  |  |  |  |
| Pr | 0.268^***^ | 0 | 0.268^***^ |
|  | -7.48 | (.) | -7.48 |
|  |  |  |  |
| Pt | -0.001 | 0 | -0.001 |
|  | (-0.09) | (.) | (-0.09) |
| **Ufo** | | | |
| Si | 0.016 | 0 | 0.016 |
|  | -1.72 | (.) | -1.72 |
|  |  |  |  |
| Ss | 0.134^***^ | 0 | 0.134^***^ |
|  | -7.01 | (.) | -7.01 |
|  |  |  |  |
| Pi | 0.108^*^ | 0 | 0.108^*^ |
|  | -2.15 | (.) | -2.15 |
|  |  |  |  |
| Pr | 0.381^***^ | 0 | 0.381^***^ |
|  | -7.3 | (.) | -7.3 |
|  |  |  |  |
| Pt | -0.019 | 0 | -0.019 |
|  | (-0.57) | (.) | (-0.57) |
| **Rtb** | | | |
| Si | 0.063 | 0 | 0.063 |
|  | -1.75 | (.) | -1.75 |
|  |  |  |  |
| Ss | 0.113^***^ | 0 | 0.113^***^ |
|  | -19.37 | (.) | -19.37 |
|  |  |  |  |
| Pi | 0.127^***^ | 0 | 0.127^***^ |
|  | -4.21 | (.) | -4.21 |
|  |  |  |  |
| Pr | 0.319^***^ | 0 | 0.319^***^ |
|  | -6.73 | (.) | -6.73 |
|  |  |  |  |
| Pt | 0.035^*^ | 0 | 0.035^*^ |
|  | -2.14 | (.) | -2.14 |
| **Rdg** | | | |
| Si | 0.059^***^ | 0 | 0.059^***^ |
|  | -6.88 | (.) | -6.88 |
|  |  |  |  |
| Ss | 0.096^***^ | 0 | 0.096^***^ |
|  | -9.92 | (.) | -9.92 |
|  |  |  |  |
| Pi | 0.117^***^ | 0 | 0.117^***^ |
|  | -9.76 | (.) | -9.76 |
|  |  |  |  |
| Pr | 0.287^***^ | 0 | 0.287^***^ |
|  | -10.42 | (.) | -10.42 |
|  |  |  |  |
| Pt | 0.009 | 0 | 0.009 |
|  | -0.6 | (.) | -0.6 |
| **Rtp** | | | |
| Si | 0.019 | 0 | 0.019 |
|  | -0.42 | (.) | -0.42 |
|  |  |  |  |
| Ss | 0.131^***^ | 0 | 0.131^***^ |
|  | -3.43 | (.) | -3.43 |
|  |  |  |  |
| Pi | -0.013 | 0 | -0.013 |
|  | (-0.49) | (.) | (-0.49) |
|  |  |  |  |
| Pr | 0.423^***^ | 0 | 0.423^***^ |
|  | -23.09 | (.) | -23.09 |
|  |  |  |  |
| Pt | 0.009 | 0 | 0.009 |
|  | -0.28 | (.) | -0.28 |
| **Rds** | | | |
| Si | 0.020^*^ | 0 | 0.020^*^ |
|  | -2.28 | (.) | -2.28 |
|  |  |  |  |
| Ss | 0.031^***^ | 0 | 0.031^***^ |
|  | -4.08 | (.) | -4.08 |
|  |  |  |  |
| Pi | 0.137^***^ | 0 | 0.137^***^ |
|  | -6.09 | (.) | -6.09 |
|  |  |  |  |
| Pr | 0.318^***^ | 0 | 0.318^***^ |
|  | -19.48 | (.) | -19.48 |
|  |  |  |  |
| Pt | 0.021 | 0 | 0.021 |
|  | -1.51 | (.) | -1.51 |
|  |  |  |  |
| *N* | 1685 |  |  |
| *t* statistics in parentheses | |  |  |
| ^*^ *p* < 0.05, ^**^ *p* < 0.01, ^***^ *p* < 0.001 | | |  |
